# The Use of Probiotic Therapy in Metabolic and Neurological Diseases

**DOI:** 10.3389/fnut.2022.887019

**Published:** 2022-05-03

**Authors:** Shirley H. F. Lee, Siti R. Ahmad, Ya C. Lim, Ihsan N. Zulkipli

**Affiliations:** Pengiran Anak Puteri Rashidah Sa’adatul Bolkiah (PAPRSB) Institute of Health Sciences, Universiti Brunei Darussalam, Gadong, Brunei

**Keywords:** therapy, microbes, obesity, probiotics, diabetes, neurodegenerative diseases

## Abstract

The human gut is home to trillions of microbes that interact with host cells to influence and contribute to body functions. The number of scientific studies focusing on the gut microbiome has exponentially increased in recent years. Studies investigating factors that may potentially affect the gut microbiome and may be used for therapeutic purposes in diseases where dysbioses in the gut microbiome have been shown are of particular interest. This review compiles current evidence available in the scientific literature on the use of probiotics to treat metabolic diseases and autism spectrum disorders (ASDs) to analyze the efficacy of probiotics in these diseases. To do this, we must first define the healthy gut microbiome before looking at the interplay between the gut microbiome and diseases, and how probiotics affect this interaction. In metabolic diseases, such as obesity and diabetes, probiotic supplementation positively impacts pathological parameters. Conversely, the gut–brain axis significantly impacts neurodevelopmental disorders such as ASDs. However, manipulating the gut microbiome and disease symptoms using probiotics has less pronounced effects on neurodevelopmental diseases. This may be due to a more complex interplay between genetics and the environment in these diseases. In conclusion, the use of microbe-based probiotic therapy may potentially have beneficial effects in ameliorating the pathology of various diseases. Validation of available data for the development of personalized treatment regimens for affected patients is still required.

## Introduction

The gut is a natural habitat for trillions of diverse microbes (anaerobic bacteria, yeasts, viruses, and bacteriophages) where the phyla Firmicutes, Bacteroidetes, and Actinobacteria are the most common (1). The gut microbiome is a complex ecosystem where microbes and their metabolites interact with host cells to influence body functions. General health is associated with a “healthy” microbiome, defined by the diversity and types of species of bacteria within the gut.

Fecal microbiome analysis has shown that the gut microbiota composition is influenced by various factors such as age, genetics, types of food consumed, economic development, and immediate environment (2–7). Dysbiosis of the microbiome is associated with a reduction in the diversity of microbes within the gut. The altered diversity of gut microbes is correlated with various diseases such as metabolic diseases, autism spectrum disorders (ASDs), and other brain disorders (8–16). Changes in the microbiome have been also linked with infection risk and susceptibility (17), including COVID-19 (18).

Recently, products containing supposedly “healthy” bacteria are touted as being beneficial to health by restoring balance to the microbiome within the gut. These products are generally termed “probiotics,” but have also generated other related products, all of which are proposed to act to enhance healthy bacteria within the gut. The term “probiotics” was coined in the 1970s while food containing beneficial bacteria have been consumed even earlier. Recently added interest in the commercialization of probiotic foods meant that there has been a need to define what can be claimed as a probiotic.

The generally accepted definition of probiotic was generated together by the Food and Agriculture Organization of the United Nations (FAO) and WHO—“live microorganisms which when administered in adequate amounts confer a health benefit on the host” (19). Other related products include “prebiotics,” “synbiotics,” “postbiotics,” and “metabiotics” (20–23). The term “live and active cultures” is sometimes used for fermented or functional foods with live microorganisms within them but those microorganisms may not prove to be probiotic yet (19).

With the plethora of probiotics and associated products now available commercially, it is no wonder that there have been many misconceptions regarding probiotics, their usage, and their health benefits, which we will address in the subsequent sections of this review.

### Constituents of Probiotics

Specific health benefits have been ascribed to particular probiotic strains, and therefore, not all probiotic supplements are equal, even if they list the same species of probiotic bacteria. Therefore, it is essential to ensure that the correct strain is used to treat the underlying clinical issue. Additionally, supplements that contain multiple strains of bacteria may also lack the scientific evidence for the claimed benefits.

The most common bacterial species used in current probiotic products are lactic acid bacteria such as *Bifidobacterium* and *Lactobacillus* strains (24). However, recent studies have identified other species of bacteria that may also confer benefits when used as probiotics, such as *Akkermansia muciniphila* and *Faecalibacterium prausnitzii* (25), and the use of these bacteria in probiotic products is rising as well (26). The effectiveness of probiotic supplementation can be measured through the bacterial load in the feces, or other measures within the body (27, 28), and is essential to establish the efficacy of any treatment.

### Evidence of Therapeutic Effects of Probiotics

The benefits of probiotic supplementation result from either inhibition of pathogen growth in the large intestine or augmented immune response and intestinal barrier function in both small and large intestines (29). As most probiotics are beneficial bacteria found naturally within the gut, ingested probiotics within the gut interact with immune cells to sustain an immunologic balance within the gastrointestinal tract (30, 31). Therefore, the interplay between the gut microbiome, probiotics, and human health is *via* the modulation of immune responses at the epithelial cells constituting the mucosal interface between host and microorganisms.

The gut microbiome also produces a wide range of metabolites due to the anaerobic fermentation of undigested materials and endogenous compounds found within the microbes and host. The metabolites produced by the microbiome serve as agents that modulate the host cells’ responses, thus its immune system and disease probability. Rooks and Garrett have reviewed how these metabolites modulate the immune responses and disease risk (32). We have summarized probiotic strains, their resulting metabolites, and their effects on health in Table 1.

**TABLE 1:** List of probiotic strains and the metabolites produced and their effects.

Probiotic (bacterial) strain(s)	Metabolites produced	Effects	References
*Bacteroides thetaiotaomicron*	Acetate	Increase mucus production	(65)
*Bacteroides thetaiotaomicron* and *Faecalibacterium prausnitzii*	Acetate and Butyrate	Ensure maintenance of appropriate secretory cells proportion	(65)
Bifidobacterium longum	Acetate	Fortifies intestinal epithelial cells integrity and prevent toxins entry into circulation	(66)
*Bifidobacterium dentium*	Acetate γ-aminobutyric acid (GABA)	Stimulates MUC2 synthesis, Promotes autophagy and calcium mobilization to release mucus	(67)
*Bifidobacterium Lactis* sp.*420*	Acetate Lactate	Modulate Cox expression profile, resulting in anti-inflammatory and anticarcinogenic properties	(68)
*Lactobacillus rhamnosus* GG and *Saccharomyces cerevisiae boulardii*	Butyrate Propionate Ethanol	Protects against pathogenic Escherichia coli	(69)
*Lactobacillus casei*	Butyrate Acetate	Increase secretion of Glucagon-like peptide-1 (GLP-1) and peptide YY (PYY) secretion	(70)
*Lactobacillus. johnsonii* L531	Butyrate Acetate Lactate	Reduces pathogen load	(71)
*Lactobacillus gasseri*	Butyrate	Exerts anti-obesity effects	(72)
*Saccharomyces boulardii*	Acetate	Antibiotic potency	(73)

Regular consumption of probiotic supplements and foods has ascribed numerous scientifically backed benefits, including effects on the gut such as amelioration of diarrhea and other digestive symptoms (33–38), reduction of inflammation (33, 39), as well as benefits to various conditions ranging from emotional imbalance to autoimmune diseases (40–45). Some groups have even shown the benefits of consuming probiotics for patients with cancer (28, 46, 47). However, it must be noted that while many clinical benefits have been rigorously tested, in many cases, probiotics cannot be considered an alternative to medicine, particularly in severe diseases.

## Healthy Gut Microbiome Profiles and Changes in Disease

Knowledge of a healthy gut microbiome is necessary before addressing the diseases triggered by the dysregulation of the gut microbiome. Hou et al. (51) established three enterotypes comprising specific species and functional composition: Bacteroides, Blautia, and Prevotella enterotypes. These different gut microbiome diversity signatures have different risks for different diseases (48–50). Additionally, the efficacy of probiotic supplementation is also affected by enterotype (51). Therefore, these enterotypes may form a basal gut microbiome that is independent of geographical location as well as nutrition.

Gut community profiles have also shown that healthy pre-adolescents have more significant numbers of species and greater diversity than adults, with increased *Firmicutes* and *Actinobacteria* (52). Both *Bacteroidetes* and *Firmicutes* bacteria are SCFAs producers, specifically acetic acid and propionic acid by *Bacteroidetes* and butyric acid by *Firmicutes* (53). Functionally, the diversity of microbial genes detected in the gut microbiome in children was responsible for the ensuing growth and development, such as vitamin synthesis. In contrast, the enriched microbial genes detected in the gut microbiome of adults are associated with inflammation and fat deposition (52). Findings from a further study to understand the gut microbiome of pre-adolescents in different geographical areas and conditions showed that the distal guts of children living in the Bangladeshi slum have significantly higher bacterial gut microbiome diversity with enrichment in *Prevotella*, *Butyrivibrio*, and *Oscillospira* together with a depletion in *Bacteroides* (54). However, this microbial diversity was more prone to changes, unlike the microbiota found in children living in the suburban community.

A reference profile comprising the abundance and list of microbes in a healthy human was constructed, with 157 organisms classified as healthy gut microbes in the Fecal Biome Population Report (55). Additionally, Kong et al. (56) studied the gut microbiome of healthy centenarians as a benchmark for a healthy microbiome model. They found that short-chain fatty acids (SCFAs)-producing bacteria were more abundant in the long-living Chinese cohort. SCFAs such as butyrate, propionate, and acetate, produced by the gut microbiome, are beneficial for health. SCFAs act by stimulating the expansion of regulatory T cells, inhibiting inflammation *via* reducing histone deacetylase-9 gene expression (57). Thus, SCFAs maintain the gut barrier’s integrity, stimulate immunity in the intestines, and prevent pathogen infection (32, 58). Hence, metabolites produced by the gut microbiome can also modulate a person’s health status (59).

Interestingly, the follow-up study revealed that the long-living healthy people in the study (both Chinese and Italian cohorts) had more diverse microbiota structures than younger age groups (60). This result contrasts with previous studies whose results have suggested that gut microbiome diversity in a person tends to decrease as the person ages (61, 62). This suggests that the changes in your gut microbiome are not set in stone and can be modulated with environmental factors and diet.

The potential of the dysbiosis of the gut microbiome in the establishment of metabolic diseases should be obvious. However, the gut microbiome is also able to communicate with the nervous system *via* the gut-brain axis (GBA) and thus affects neurological diseases as well. The GBA involves bidirectional interaction between the central and the enteric nervous systems, connecting the cognitive and emotional centers of the brain with peripheral intestinal functions. Bacteria in the gastrointestinal (GI) tract influence the signaling of neural pathways and the central nervous system (CNS) (63–67). Evidence of microbiota-GBA communications emerged from the association of dysbiosis with central nervous disorders (63, 68, 69). From this, we note that healthy gut microbiota is essential for brain development and function.

Consequently, a healthy gut microbiome is essential for both metabolic and neurological health. In the following sub-sections, we will be addressing the use of probiotics in metabolic diseases (obesity and type II diabetes) and neurodegenerative diseases.

### Gut Dysbiosis and Probiotics and Obesity

Obesity is defined by excessive fat accumulation in the body, which may increase the risk of non-communicable diseases such as diabetes, cardiovascular diseases, some cancers, and hypertension (70). The gut microbiome and the composition of dietary intake are profoundly linked (71). For example, the intake of animal-based foods provided up to 5 consecutive days of increased bile-tolerant microbes (*Alistipes*, *Bilophila*, and *Bacteroides*) and reduced the amount of fiber-fermenting bacteria (72).

Gut microbiota profiles in overweight and obese individuals show higher amounts of *Bacteroides, Bifidobacteria, Staphylococcus aureus*, and *Lactobacilli Clostridia* (73, 74). Among overweight individuals, the baseline ratio of gut microflora, *Firmicutes* to *Bacteroidetes* was disturbed (75). *Firmicutes* bacteria potentially are able to affect the modulation of gene expression and hormones involved in metabolism (76). Therefore, the change in the ratio of different bacteria species may affect human metabolism, leading to obesity.

Probiotics may act as anti-obesity agents by various modes of action, including modulation of specific gut microbiota strains, gastrointestinal and immune system modulation, lowering insulin resistance, and greater satiety. The use of probiotics containing *Lactobacillus* and *Bifidobacterium* species in obesity treatment is promising (77). Some of the positive changes which resulted from the intake of probiotics include lower waist circumference, lower body fat deposition, lower body weight, lower weight gain, and improved lipid profile. However, Vajro et al. showed that *L. salivalis* supplementation in obese adolescents led to no improvement in obesity parameters (78). Another study with the consumption of one capsule of *L. rhamnosus* G showed a lower weight gain at 1 year of life and up to 4 years old in children but observed no weight changes after that period (79). This difference in weight gain patterns may be due to the colonization of the gut microflora, which begins during the first few years of life (80, 81). Unless various scientific groups consistently match the age of controls and subjects, together with consistent bacterial strains utilized in probiotics, the conclusion derived from the comparison of these studies remains murky.

*A. muciniphila* is negatively correlated to obesity development, as well as other diseases such as type-2 diabetes and hypertension (82). A human clinical trial looking at the impact of *A. muciniphila* supplementation for over 3 months showed that the treatment led to improved insulin sensitivity, insulinemic, and reduction of total cholesterol (83). The evidence of *A. muciniphila* as a probiotic that confers a protective effect against metabolic disorders has been accumulating over the past few years (84) and may merit further study.

Hence, probiotics positively impact the reduction of relevant obesity parameters, although the effect varies across the different age groups and genders. More standardized studies are needed to investigate how the different mixtures of bacterial species in probiotics affect different age groups and genders.

### Gut Dysbiosis, Probiotics, and Diabetes

Type-2 diabetes is a metabolic disorder in which individuals display abnormally high blood glucose, resulting from inadequate insulin secretion and resistance (85). Type 2 diabetes results from the interaction between environmental factors and genetic factors (86). One of the primary risk factors of type-2 diabetes is being overweight or obese (87).

A change in the composition of the gut microbiota may result in increased susceptibility to prediabetic conditions such as insulin resistance (87–89). Reports have revealed that the intestinal microbiome of individuals with type-2 diabetes has reduced butyrate-producing bacteria (87, 90), a lower frequency of Firmicutes, and a higher frequency of Bacteroidetes and Proteobacteria (88). The metabolites produced by gut microbes also affect insulin sensitivity and glucose homeostasis, with metabolites like SCFA improving insulin secretion (91). Therefore, butyrate-producing bacteria affect insulin secretion and therefore, the blood sugar level of a person. Further exploration of the bacterial strain or administration of butyrate may be beneficial to a diabetic.

Probiotic intake, such as *Lactobacillus rhamnosus* GG, leads to improvement in intestinal integrity, reduced lipopolysaccharide level, reduced endoplasmic reticulum stress, and improved insulin sensitivity (91–93). Animal and clinical trials have shown that both single probiotic strains or mixtures of probiotics have the potential to improve type-2 diabetes parameters (87, 94). More research is required to dissect the most suitable species impacting gut metabolism, as well as exposure time, and dose.

### Gut Dysbiosis, Probiotics, and Autism Spectrum Disorder

Autism spectrum disorder is a group of neurodevelopmental disorders defined by deficits in communication and social interaction, and stereotyped behaviors (65). GI abnormalities are common among individuals with ASD (95, 96), with a strong correlation of GI symptoms with ASD severity (97).

The gut microbiota of children with ASD is less diverse, with decreased levels of health-promoting gut bacteria, and an increased abundance of species that produce neurotoxins (65). Metabolites from the gut microbiota may play vital roles in the pathogenesis of ASD (95, 96). Altered fecal SCFAs have been linked to constipation in ASD (97), where lower levels of acetic acid and butyrate and an elevated level of valeric acid have been reported in subjects with ASD (96). It has also been shown that SCFAs can induce autistic-like symptoms upon injection into rats (98).

Maternal immune activation (MIA) mouse models that display features of ASD have altered microbiota and GI barrier defects. Oral treatment of MIA offspring with the human commensal bacteria *Bacteroides fragilis* improves gut permeability, alters the microbial composition, and corrects behavioral defects in MIA animals. Therefore, it has been proposed that targeting the gut microbiota may be a potential therapy for specific symptoms in ASDs (95).

Probiotics potentially impact gut microbiota communities to alter the levels of harmful metabolites in ASD children, reducing GI inflammation and intestinal permeability (1, 99). However, the results of probiotic supplementation in individuals with ASD remain inconclusive and controversial. Current probiotics are mainly aerobic, short-lived, milk-derived cultures, which are not usually a significant part of the primarily anaerobic human gut microbiome (1). A review based on four studies concluded that current evidence does not support the use of probiotics to modify behavior in patients with ASD (100). Probiotics did not exert a significant effect to restore most of the beneficial bacteria upon assessment of stool samples from 58 individuals with ASD and 39 age-matched typically developing children (97). On the other hand, it has also been reported that probiotics treatment seems to improve ASD-associated behavioral symptoms (101).

Autism spectrum disorder individuals are highly selective eaters (102, 103); therefore dietary factors remain a strong confounding factor for these individuals. The complex interplay between host genetics, environment, and the microbiome although challenging to dissect are important factors to consider. Larger longitudinal trials as well as optimizing dosage, formulation (single vs. multispecies probiotics), timing (101), route of administration as well as toxicity concerns remain to be addressed to validate the efficacy of probiotics for ASD, taking into consideration age and population-specific differences in gut microbiota/metabolites produced (6, 7).

### Gut Dysbiosis and Probiotics in Neurodegenerative Diseases

It is well-established that age is a primary risk factor for neurodegenerative diseases due to increased insults including decreased neurotransmitter levels, chronic inflammation, oxidative stress, and apoptosis (104). There is also a high prevalence of GI comorbidities among patients with Parkinson’s and Alzheimer’s diseases (105, 106). Dysbiosis in the intestinal microbiota in the elderly may result in a leaky gut, and subsequently, promote systemic and neuroinflammation (107).

Gut microbiota secretes neurometabolites, which include neurotransmitters that regulate the signaling cascades of the CNS. A comprehensive review of neurotransmitters directly secreted by various probiotics has been published (105). Altered levels of neurotransmitters result in behavioral changes in neurodegenerative diseases. Restoring the balance of neurotransmitters by targeting gut microbiota is therefore central to the management of neurodegenerative disease.

Parkinson’s disease (PD) is characterized by loss of dopaminergic neurons and intraneuronal alpha-synuclein accumulation, in the basal ganglia and at peripheral sites, including the gut (108). GI dysfunction has been reported to be a potential contributor to the pathogenesis of PD with evidence that alpha-synuclein inclusions appear early in the enteric nervous system and travel to the brain *via* the vagal nerves (109, 110). A review on altered gut microbiota compositions in patients with PD is available (111). Probiotics administration in independent studies improves GI symptoms and the metabolic profile of patients with PD (108, 111, 112).

Alzheimer’s disease (AD) is one of the most common irreversible, neurodegenerative disorders in the elderly, which leads to cognitive decline and dementia. Inflammatory response at the site of beta-amyloid (one of the hallmark features of AD) accumulation in the brain has been linked to the gut microbiota (66). Current studies on the efficacy of probiotics in AD, although limited, seems promising. In a transgenic mice model of Alzheimer’s Disease (AD), modulation of the gut microbiota through exercise and probiotic treatment alleviated the progress of AD (113). Rats injected with probiotics (*L. acidophilus, L. fermentum, B. lactis, B. longum*) for 8 weeks elicit an improvement in memory deficit and AD-associated pathology (114). However, it remains to be determined whether these findings are replicable in humans. Another randomized, double-blind, and controlled clinical trial among 60 patients with AD revealed that a 12-week probiotic (*L. acidophilus, L. casei, B. bifidum*, and *L. fermentum*) consumption improved cognitive function and certain metabolic markers (115). There is also an ongoing clinical trial (randomized, placebo-controlled) to investigate the effect of probiotics on 58 participants with dementia (116). Therefore, the efficacy of probiotics to restore gut dysbiosis in patients with AD awaits further validation.

A key limitation of current probiotic studies for PD and AD is the small sample sizes (*n* < 100). Consistent study designs in larger human trials with validated safety and efficacy are required before translation into clinical settings.

## Future Directions

Manipulation of the gut microbiota and microbial metabolites to address challenging questions in metabolic and brain disorders is difficult due to the complex relationship between host genetics and environmental factors to influence the gut microbiota. A healthy diet and exercise positively modify the gut microbiota (117–119), therefore it remains inevitable to tackle these key modifiable factors to ensure a healthy community of microbes.

Utilizing data from the NIH Human Microbiome Project (HMP) for resources and insights on the human microbiome provides an opportunity to further understand the complex relationship between human health and diseases, which will serve as a pedestal for novel approaches toward the development of therapeutics to tackle relevant diseases. Large scale, harmonized multi-center studies, and freely accessible data are imperative to validate the role of probiotics as potential therapeutics before translating research into clinical practice.

The long-term effects of probiotics and their corresponding metabolites/substances on health are needed to fully understand the mechanisms of each probiotic strain on health (120). Delineation of the precise role and effect of each probiotic strain may just be the beginning of introducing precise probiotic strain for an exact clinical disease. This delineation may be followed by combined efforts of various strains of probiotics. In short, the journey into the gut microbiome is just the tip of the iceberg at the moment.

## Author Contributions

IZ provided the concept of the manuscript and finalized the manuscript. All authors wrote, provided revisions to the manuscript, read and approved the final manuscript, reviewed the manuscript, and consented for it to be sent for publication.

## Conflict of Interest

The authors declare that the research was conducted in the absence of any commercial or financial relationships that could be construed as a potential conflict of interest.

## Publisher’s Note

All claims expressed in this article are solely those of the authors and do not necessarily represent those of their affiliated organizations, or those of the publisher, the editors and the reviewers. Any product that may be evaluated in this article, or claim that may be made by its manufacturer, is not guaranteed or endorsed by the publisher.
